# Barriers and facilitators to use of non-pharmacological treatments in chronic pain

**DOI:** 10.1186/s12875-017-0608-2

**Published:** 2017-03-20

**Authors:** William C. Becker, Lindsey Dorflinger, Sara N. Edmond, Leila Islam, Alicia A. Heapy, Liana Fraenkel

**Affiliations:** 1 West Haven VA Medical Center, VA Connecticut Healthcare System, Mail Stop 151B 950 Campbell Avenue, West Haven, CT 06516 USA; 2 Yale University School of Medicine, New Haven, CT USA; 3 Walter Reed National Military Medical Center, Bethesda, MD USA; 4 University of Maryland School of Medicine, Baltimore, MD USA

**Keywords:** Chronic pain, Non-pharmacological treatment, Qualitative research

## Abstract

**Background:**

Consensus guidelines recommend multi-modal chronic pain treatment with increased uptake of non-pharmacological pain treatment modalities (NPMs). We aimed to identify the barriers and facilitators to uptake of evidence-based NPMs from the perspectives of patients, nurses and primary care providers (PCPs).

**Methods:**

We convened eight separate groups and engaged each in a Nominal Group Technique (NGT) in which participants: (1) created an individual list of barriers (and, in a subsequent round, facilitators) to uptake of NPMs; (2) compiled a group list from the individual lists; and (3) anonymously voted on the top three most important barriers and facilitators. In a separate process, research staff reviewed each group’s responses and categorized them based on staff consensus.

**Results:**

Overall, 26 patients (14 women) with chronic pain participated; their mean age was 55. Overall, 14 nurses and 12 PCPs participated. Seven healthcare professionals were men and 19 were women; the mean age was 45. We categorized barriers and facilitators as related to access, patient-provider interaction, treatment beliefs and support. Top-ranked patient-reported barriers included high cost, transportation problems and low motivation, while top-ranked facilitators included availability of a wider array of NPMs and a team-based approach that included follow-up. Top-ranked provider-reported barriers included inability to promote NPMs once opioid therapy was started and patient skepticism about efficacy of NPMs, while top-ranked facilitators included promotion of a facility-wide treatment philosophy and increased patient knowledge about risks and benefits of NPMs.

**Conclusions:**

In a multi-stakeholder qualitative study using NGT, we found a diverse array of potentially modifiable barriers and facilitators to NPM uptake that may serve as important targets for program development.

## Background

The landmark 2011 Institute of Medicine report on pain care in the U.S. highlighted that multimodal, biopsychosocially-oriented treatment that promotes patients’ self-management skills is the optimal paradigm for improving the effectiveness of chronic pain treatment [1]. However, the report noted that chronic pain treatment is frequently solely pharmacologic and excludes evidence-based non-pharmacological pain treatment modalities (NPMs) [2]. With mounting evidence that treatment relying solely on pharmacotherapy is often unsafe and/or ineffective in chronic pain treatment, consensus recommendations increasingly promote a multi-modal treatment strategy [1, 3, 4]. This strategy seeks to shift the clinical paradigm away from heavy reliance on medications to a treatment approach that incorporates a diverse array of NPMs targeting the complex nature of chronic pain and promotes patient self-management [5, 6].

Multiple studies have demonstrated the effectiveness of NPMs in improving chronic pain outcomes, including, for example, physical therapy [7–9], cognitive behavioral therapy [10–12], mindfulness-based stress reduction [13], yoga [14–16], and chiropractic treatment [9]. While medication-only treatment strategies may foster passive coping styles, NPMs’ benefits may be realized in part through a reinforcing cycle of patient self-efficacy, active problem-solving, realistic goal setting and a functional/rehabilitative outlook [17, 18].

Because of the widespread prevalence of chronic pain and the major impact it has on quality of life, integrated health systems such as Kaiser Permanente and the Veterans Health Administration (VHA) have sought to make multi-modal pain care widely available [19], even establishing virtual treatment networks relying on telehealth to deliver some NPMs to remote areas [20]. Despite these efforts, at some centers, NPM utilization remains relatively low [21]. In response, the Institute of Medicine, and more recently the Department of Health and Human Services, called for a comprehensive examination of barriers “to help close the gap between empirical evidence regarding the efficacy of pain treatments and current practice.” [1] In an effort to identify such barriers and facilitators to ultimately inform the design of effective strategies for health systems to increase utilization of NPMs, we studied the perspectives of two stakeholder groups: patients with chronic pain and healthcare professionals (nurses and primary care providers (PCPs)). While other studies have examined qualitative factors related to pain management from patient [22, 23] and provider perspectives [24–26], our study is novel in its focus on non-pharmacological treatments, examining patient and provider perspectives simultaneously, our use of the nominal group technique (described below), and our inclusion of nurses, whose role in delivering multimodal, team-based pain care is essential.

## Methods

### Overview

Because our aim was more to identify themes than interpret perspectives, we employed qualitative description methodology with thematic analysis to study the question what are the consensus-based most-important barriers and facilitators to greater uptake of NPMs for chronic pain? To obtain data for the study, we convened eight separate groups of participants and engaged them each in a Nominal Group Technique (NGT) process. The NGT process, described in detail below, allows researchers to generate consensus among stakeholders regarding answers to focused questions [27, 28]. We viewed NGT as an attractive data gathering approach for a number of reasons: 1) it encourages balanced participation among participants; 2) it offers the opportunity to generate a breadth of factors, albeit potentially sacrificing depth; and 3) it brings with it group consensus and closure that may be lacking in other group methods [29].

### Participants

Most of the included patients were recruited by their PCPs as having previously shared opinions on pain treatment; a small minority we recruited through a flyer. Patients were age 70 or younger with an average numeric pain rating scale score of 4 or higher on most days of the past month as ascertained on phone screening by a research assistant. Patients needed to be fluent in English and cognitively intact, ascertained in the screening phone call; psychiatrically and medically stable, determined by the absence of inpatient admissions in the prior 30 days; and lack cancer diagnoses documented by electronic health record review. Patients received $20 for participation. We recruited nurses and PCPs at staff meetings and via email as PCPs and primary care nurses in VA settings are the first line of treatment for chronic pain. They are also responsible for the vast majority of referrals to NPM services. There were no eligibility criteria for healthcare professionals beyond employment in the setting and direct provision of patient care. Those who agreed to participate generally considered pain treatment an important topic for discussion.

### Study design and NGT description

We convened eight separate nominal groups: four patient groups (two with women only and two with men only), two nurse groups and two PCP groups. We separated patient groups by sex to ensure comfort participating in groups; at our center, as well as other VHA medical centers, women have separate primary care clinics and we sought to achieve a similar environment. Each group, consisting of the recommended 5–9 subjects [30], participated in an NGT session facilitated by members of the research team (authors WCB, LD and LI). After providing a brief background of the study and explanation of terms and procedures to orient participants, we asked participants about barriers to NPM uptake as follows: “What are some barriers to patients using non-pharmacologic pain treatments? In other words, why don’t some patients use these kinds of treatments or what makes it harder for patients to use them?” We showed all groups a picture card depicting five specific NPMs and briefly described each one: physical therapy, cognitive behavioral therapy, yoga, chiropractic and mindfulness based stress-reduction. After completing the full NGT process described below and a 1–2 min break, we then asked about facilitators as follows: “What are some of the things that make it more likely for patients to use non-pharmacologic pain treatments? What makes it easier to use these kinds of treatments?”

In each round, we asked participants to silently write down as many responses to the question as possible in five minutes. After this, a researcher asked each participant to read one answer aloud in a round robin fashion, while another researcher wrote the responses on a flip chart without discussion or editorializing. Once all answers were on the flip chart, we engaged the group in discussion for the purposes of clarifying any of the responses, editing as necessary, and consolidation of very similar or identical answers, as judged by the participants. Once the final list of consolidated answers was complete for “barriers,” each participant anonymously voted on a most important, 2nd most important and 3rd most important response by writing her or his votes on a note card. The same sequence of processes was repeated for “facilitators.” We did not instruct participants to link facilitators to the barriers they provided in the first round.

### Data analysis

We collected basic demographic information on participants to provide a description of the sample. Following standard methodology [31], for each nominal group, we tallied voting points for each of the barriers and facilitators identified: “Most important” votes received 3 points; “2^nd^ most important” received two points and “3^rd^ most important” received one point. The tallied points allowed for a group-level ranking of responses. To facilitate comparisons across groups and interpretation of the findings, the research team used thematic analysis to categorize responses based on our consensus interpretation of their meaning. Since several individual nominal groups listed over 20 barriers and facilitators, but there was striking similarity in responses between similarly composed groups, we combined both women groups’, men groups’, nurse groups’ and PCP groups’ responses.

## Results

### Participant characteristics

Overall, data was collected from 52 participants: 26 patients and 26 healthcare professionals. Demographic and clinical data on the participants is presented in Table 1.

**Table 1:** Participant Characteristics

Patients (*N* = 26)	Healthcare professionals (*N* = 26)
Women: 14	Women: 19
Men: 12	Men: 7
Age, years (mean): 55	Age, years (mean): 45
Causasian: 12	Causasian: 18
African-American: 7	African-American: 2
Hispanic: 3	Hispanic: 2
American Indian: 1	Asian: 2
Other: 3	Other: 2
Tried NPM in past year: 20	
Current opioid therapy: 12	

### Response categories

We identified five categories of responses present across participant groups: (1) access; (2) awareness or knowledge; (3) patient-provider interaction; (4) treatment beliefs; and (5) support. Table 2 displays barriers and facilitators, by category, highlighting the highest rated factors by participant groups.

**Table 2:** Factors related to uptake of non-pharmacological pain treatment modalities (NPMs)

Category	Barriers	Facilitators
Access	Transportation •Distance to travel^3F,3N^ •Cost of travel •Lack of transportation Scheduling •Delays in NPM appointments •Low availability of appointments •Time of day services offered is not convenient Out-of-pocket cost •High cost (of treatment, travel or equipment) or not covered by insurance^1F^ •Lack of insurance coverage Resources •Some NPMs not available at VA^1M^ •Not having equipment to do at home •Most programs are male-focused •Not having ability to continue treatment because of limited number of sessions •Lack of delivery of NPM in the format patient prefers (e.g., web based)	Transportation •NPM sessions closer to home^2F,2N^ •Covered mileage/pay for mileage •Transportation assistance/voucher •Fewer visits to the VA Scheduling •Shorten wait time for NPMs •Patients access treatments directly/self-referral •Scheduling flexibility for NPM sessions, including evening/weekend appointments •Consistent treatment schedule •Quick/easy access to NPMs^3P^ •Patient leaves primary care visit with appointment for NPM •Easy-to-use consult template in the electronic record Out-of-pocket cost •No cost/low cost/covered by insurance Resources •Wider variety of NPMs readily available^1F^ •Use non-VA treaters/contract services out •Female only treatment sessions •Ability to practice NPM while at appointment •Ability to have appointments in more private settings to reduce stigma •Treatments more individualized •Sufficient staff in the specialty services to deliver NPMs •Techniques patients can do own their own; self-management
Awareness or knowledge	•Both patients and providers not being sure what the NPM entails or rationale for it^2N^ •Both patients’ and providers’ lack of knowledge of which NPMs are available •NPMs not advertised on television •Patients’ poor understanding of pain	•Better explanation of what to expect and rationale for treatments^3F, 1M^ •Better advertising that these services are offered^3N^ •Increase patient knowledge about risks and benefits of NPMs^1N^ •Educating staff/providers about treatment availability •Word of mouth from other Veterans •Increase staff/provider knowledge about risks and benefits of NPMs •Show providers the evidence/guidelines for choosing appropriate NPMs •Educate the patient about nature of chronic pain
Patient-provider interaction	•Patient’s lack of motivation^2M, 2F^ •Difficult for patient to advocate for NPMs they would prefer •Stigma of condition/having to explain it at NPM sessions •Providers not believing pain; suggesting NPMs is evidence of that •Disliking NPM provider •Providers not believing pain •Patients’ distrust that the referring provider is offering best plan •Patient perception that NPM providers are “quacks” •Patients with history of physical abuse don’t want to use NPMs •Many women don’t like to be touched and many NPMs are hands on	•Empathy/compassion from provider •Ready state of mind (patient) •Respect for patients’ input on plan •Patients’ good rapport with PCPs •Open communication between PCP and patient •Good personal qualities of the NPM providers •Well thought out treatment plan specific to the patient •Trusted provider promoting NPMs/multimodal care •Shared decision-making regarding components of treatment plan
Treatment beliefs	Perceived lack of efficacy of NPMs •Patient skepticism about efficacy of NPMs^1P^ •Fear that treatment will fail •Lack of commitment to treatment •Elderly veterans don’t believe in “new age” treatments •Provider and patient skepticism about efficacy of NPMs •Patients’ perceptions of the VA as offering lower quality of care and NPMs are viewed in that same light Perceived burden of NPMs •Pain, stress or other physical conditions prevent people from engaging with NPMs^3M^ •Lack of patient motivation/energy •Time commitment •Patients’ perception that they’re in too much pain to do some NPMs •Long course of treatment requires a significant time commitment •Patients’ preference to avoid self-care/self- management •Expectation from patient of getting help (instead of having to do something active) •Needing time off from work to attend treatment with multiple visits Perceived harm of NPMs •Patients’ fear of adverse effects or injury •Patients’ fear of pain getting worse with NPMs •Expectation from patient of an opioid prescription; NPMs seen as substandard Medication-related •Perception among patients that medications are more effective^1N^ •Previously formed opinions based on culture/ads: Need a magic pill^2P^ •Opioids have been used and worked^3P^ •Easier to take a pill	Perceived efficacy of NPMs •Perception among patients that medications are more effective^1N^ •Previously formed opinions based on culture/ads: Need a magic pill^2P^ •Opioids have been used and worked^3P^ •Reinforce positive NPM-related beliefs (e.g. NPMs may have less adverse effects than medications; NPMs promote an active lifestyle; NPMs may help you find other ways to do things you enjoy) Perceived safety of NPMs •Comfort with the NPM provider/being touched Medication-related •If patient wants to be on fewer pills/recognize NPMs are a healthier way than consuming chemicals^3M^ •If doctor won’t prescribe narcotics •Not stopping pain medications all at once Other •Mental health treatment involvement •Elicit expectations of pain treatment and pain level patient can live with
Support	Social •Lack of patient support from family and doctor •Clinicians not offering positive reinforcement •Lack of positive family or friend influence •Lack of community or club that does NPMs	Healthcare system •Encouragement from team that is trained, positive, and willing to work with you^2M^ •Have a coach •Group support •Support beyond the initial discussion; e.g. follow up to boost/maintain motivation •Collaboration/trust between PCPs and specialists •Specialty staff being friendly, engaging, rapport- building Social •Supportive family •Encourage family participation •Encourage peer support

1 = highest scored factor, 2 = second highest scored factor, 3 = third highest scored factor

M = male patient group, F = female patient group, N = nurse group, P = primary care provider group

### Barriers

Several barriers related to access were identified, including factors related to transportation, scheduling, out-of-pocket costs, and resources. Patients rated distance to travel, high cost of treatment, and lack of some NPM availability as important access barriers; each of these barriers was also mentioned by providers. The highest rated access barrier by providers was the travel required by patients.

Regarding barriers related to NPM awareness or knowledge, providers acknowledged that both patients and providers are unsure of what some NPMs entail or the rationale for NPMs. Patients agreed that a lack of knowledge on the rationale for treatment was a barrier. Further, both patients and providers reported being unaware of what NPMs are available.

A range of barriers related to patient-provider interactions were identified. Provider groups mentioned patient trust in PCPs, patient perceptions of NPM providers, and personal preferences regarding treatment modalities as barriers. The most important patient-identified barrier in this domain was patient’s lack of motivation.

Beliefs about treatment were identified as barriers to NPM use. Medication-related beliefs such as patient perception that medications are more effective were identified as one of the most important barriers reported by providers. Skepticism about the efficacy for NPMs by both patients and providers was identified as a barrier, including patient and provider beliefs that NPMs are not effective, that NPMs will fail, or that NPMs are substandard treatment (i.e., as compared to medications). The burden of NPMs, including the long course of treatment, the time commitment required, and the perceived pain or stress that may accompany engagement in NPMs were also identified, as were concerns about the potential harm of NPMs (e.g. worsening pain, exacerbation of health problems).

Finally, support (or lack thereof) from the healthcare system and from one’s social support system were identified as potential barriers. For example, providers noted a lack of positive influence from family or friends, while patients noted a lack of patient support from families and from doctors.

### Facilitators

After reviewing barriers, participants were asked to brainstorm potential facilitators that may help patients engage in NPMs. Regarding access, having NPM sessions closer to home (or in the home) was rated highly important by both patients and providers. Other facilitators included having a wider variety of NPMs readily available and ensuring timely and easy access to NPMs.

To enhance awareness and knowledge of NPMs, patients highly rated the need for better explanations of what to expect and a better rationale for NPM treatments; similarly, providers rated increased patient knowledge and better advertising about services offered as important. In addition to patient education, both groups mentioned educating providers about the evidence for and availability of NPMs.

Patients identified several facilitators related to patient-provider interaction, including provider empathy, respect for patients’ preferences and open communication. Providers identified empathy and compassion, as well as shared decision making, as potential facilitators. Similar, within the domain of support, patients noted that support and encouragement from the medical team was an important facilitator.

Providers identified several facilitators related to treatment beliefs, including patient belief in the efficacy of NPM treatment and the belief that NPM recommendations are part of a standard protocol. Patients and providers both noted that reinforcing positive NPM-related beliefs, such as the belief that NPMs can be effective and may have fewer adverse side effects than medications, were important.

## Discussion

This multi-stakeholder qualitative study on barriers and facilitators to use of NPMs for chronic pain elicited a wide array of patient-, provider- and systems-related factors that likely contribute to use and non-use of these evidence-based treatments. These factors, which we categorized as related to access, patient-provider interaction, treatment beliefs, and support, represent a number of important targets for implementation efforts locally and may generalize to other populations and health systems. Overall, patients and providers identified very similar barriers and generated many of the same facilitators, demonstrating consistency in beliefs about NPM use among various stakeholder groups. As chronic pain is a highly prevalent and costly condition, we focus below on potential interventions, discussed by category, that have broad applicability and relevance.

Particularly from the patients’ perspectives, barriers related to access to NPMs— transportation, cost, scheduling and resources—were especially prominent. VHA’s system-wide access challenges have been in the spotlight recently [32]; some of the proposed solutions to these broader access issues were echoed in this study. For example, sub-contracting pain treatment services to private facilities closer to patients’ homes may leverage transportation cost savings to offset increased treatment expense for the health system. Technology may also play a role in enhancing access as web-based and telehealth platforms for delivering NPMs such as cognitive behavioral therapy continue to advance [33]. In-person treatments using group formats—for example yoga [34], structured exercise classes, chronic pain schools, and mindfulness-based stress reduction groups [26]--can expand treatment capacity. Offering group sessions outside of typical work hours may be another effective approach to expanding access.

Awareness and knowledge-related factors as well as treatment belief-related factors revealed a number of fundamental issues that, considered together, suggested a need for a broad-based, multi-pronged implementation strategy. A primary concern was the perception that referring providers and patients alike are skeptical about NPMs, do not understand the rationale for NPMs, nor do they know what many NPMs entail. Academic detailing, in which providers are educated about treatment strategies [35] could be one approach to enhancing education; however, our findings suggest that targeting provider education alone would not be sufficient since patients’ attitudes and preferences were also identified as barriers, suggesting that provider and staff training in communication and education (of patients) about the multimodal pain treatment philosophy is needed.

Furthermore, patients and providers’ lack of awareness of NPMs’ availability suggested the need for advertising campaigns – perhaps using novel methods such as social media. A broad-based promotion of the multimodal treatment paradigm, reflecting an institutional belief in and commitment to the treatment philosophy, may help support culture change. Australia’s “Back Pain: Don’t Take It Lying Down” campaign is one such successful example [36]. Several inaccurate but commonly held treatment beliefs – for example, that NPMs cannot or should not be used if patients are experiencing stress or other significant medical issues – could be specific targets for motivational enhancement and educational messages.

Embedded in addressing treatment beliefs and increasing knowledge and awareness of NPMs is the need to improve patient-provider interactions. Distrust in providers, the belief that referral to NPMs occurs because pain is not believed, and patient’s lack of motivation to engage in NPMs all suggest that training providers in more effective communication is important. The use of motivational interviewing strategies as well as other pain communication strategies such as validation [37], are needed to help providers more effectively engage with patients with chronic pain. Similarly, lack of support from medical providers, peers, friends, and family was identified as a potential barrier to NPM utilization, suggesting that support is needed for successful engagement in NPM treatments. Indeed, encouragement from the medical team was identified as one of the most important facilitators to NPM engagement.

It is also clear from our findings that while we explicitly focused on NPMs, educational and clinical interventions must consider the role of pharmacologic treatments, especially opioids, when educating patients and providers about pain. Primary care providers, most often the prescribers of opioids for chronic pain in VHA, expressed frustration about the lack of tools for communicating with patients receiving opioids about the importance of NPMs and lack of support in follow up for patients around this issue. Scripted messaging from providers about the relative efficacy of NPMs compared to opioids—that, in fact, NPMs show at least equivalent and perhaps superior benefit—may increase acceptance of NPMs. Also, expert recommendations strongly support NPMs in conjunction with long-term opioid therapy [3], suggesting engagement with NPMs could be considered a pre-requisite for ongoing opioid therapy as part of treatment agreements. Evidence-based collaborative care models [38, 39] in which nurses or other mid-level providers follow up on multimodal pain treatment plans to assess barriers to adherence and enhance patient motivation may be critical to re-distributing work load away from PCPs and improving quality of care. These models are also consistent with patient report that continued encouragement from their care team would be a significant facilitator to NPM engagement. This last point also related to the factor of support, another recurring theme in our data. Besides care management and structured follow up, our findings suggested peer and family support interventions as other potentially effective strategies, models well-supported in the treatment of other chronic conditions [40, 41].

Strengths of the study included the large and diverse group of participants from two stakeholder groups, increasing the likelihood that important barriers and facilitators were not missed. The NGT methodology itself also contributed to this strength since it encourages active involvement of all participants. This study has limitations. The nominal groups were performed in one integrated health system with a relatively robust array of NPMs; the barriers and facilitators identified may not be generalizable to other settings. Furthermore, many patients reported some use of NPMs in the recent past; a different sample of patients with less experience with NPMs may have identified different kinds of barriers and facilitators. Also, because the drawbacks of long-term opioid therapy and calls for renewed focus on NPMs have dominated discourse in the U.S., we asked participants to consider all NPMs as a singular group; however, asking participants to consider different kinds of NPMs as a homogenous group may have obscured important barriers and facilitators to uptake of specific NPMs. Finally, participants’ responses to the study questions may have been subject to bias, including social desirability bias. To mitigate this possibility, participants were asked to consider not only their own perspectives, but perspectives of others they may have heard about.

## Conclusions

In this large qualitative study of barriers and facilitators to use of NPMs for chronic pain, the inclusion of multiple stakeholder groups led to a robust array of factors that could serve as targets for developing interventions aimed at improving uptake of these evidence-based treatments.

## Abbreviation

NGT: Nominal Group Technique
NPMs: Non-pharmacological pain treatment modalities
PCPs: Primary care providers
VHA: Veterans Health Administration

## References

[1] Committee on Advancing Pain Research Care, Institute of Medicine: Relieving pain in America: A blueprint for transforming prevention, care, education, and research. Washington: National Academies Press; 2011. 22553896

[2] Mafi JN, McCarthy EP, Davis RB, Landon BE (2013). Worsening trends in the management and treatment of back pain. JAMA Intern Med.

[3] Chou R, Fanciullo GJ, Fine PG, Adler JA, Ballantyne JC, Davies P, Donovan MI, Fishbain DA, Foley KM, Fudin J (2009). Clinical guidelines for the use of chronic opioid therapy in chronic noncancer pain. J Pain.

[4] The Management for Opioid Therapy for Chronic Pain Working Group: VA/DoD Clinical Practice Guideline for Management of Opioid Therapy for Chronic Pain. 2.0 edn. Washington: Office of Quality and Performance; 2010.

[5] DeBar L, Kindler L, Keefe F, Green C, Smith D, Deyo R, Ames K, Feldstein A (2012). A primary care-based interdisciplinary team approach to the treatment of chronic pain utilizing a pragmatic clinical trials framework. Transl Behav Med.

[6] Turk DC, Wilson HD, Cahana A (2011). Treatment of chronic non-cancer pain. Lancet.

[7] Hill JC, Whitehurst DG, Lewis M, Bryan S, Dunn KM, Foster NE, Konstantinou K, Main CJ, Mason E, Somerville S (2011). Comparison of stratified primary care management for low back pain with current best practice (STarT Back): a randomised controlled trial. Lancet.

[8] Ferreira ML, Ferreira PH, Latimer J, Herbert RD, Hodges PW, Jennings MD, Maher CG, Refshauge KM (2007). Comparison of general exercise, motor control exercise and spinal manipulative therapy for chronic low back pain: a randomized trial. Pain.

[9] Cherkin DC, Deyo RA, Battié M, Street J, Barlow W (1998). A comparison of physical therapy, chiropractic manipulation, and provision of an educational booklet for the treatment of patients with low back pain. N Engl J Med.

[10] Lamb SE, Hansen Z, Lall R, Castelnuovo E, Withers EJ, Nichols V, Potter R, Underwood MR (2010). Group cognitive behavioural treatment for low-back pain in primary care: a randomised controlled trial and cost-effectiveness analysis. Lancet.

[11] Wetherell JL, Afari N, Rutledge T, Sorrell JT, Stoddard JA, Petkus AJ, Solomon BC, Lehman DH, Liu L, Lang AJ (2011). A randomized, controlled trial of acceptance and commitment therapy and cognitive-behavioral therapy for chronic pain. Pain.

[12] Keefe FJ, Caldwell DS, Williams DA, Gil KM, Mitchell D, Robertson C, Martinez S, Nunley J, Beckham JC, Crisson JE (1991). Pain coping skills training in the management of osteoarthritic knee pain: a comparative study. Behav Ther.

[13] Cherkin DC, Sherman KJ, Balderson BH, Cook AJ, Anderson ML, Hawkes RJ, Hansen KE, Turner JA (2016). Effect of mindfulness-based stress reduction vs cognitive behavioral therapy or usual care on back pain and functional limitations in adults with chronic Low back pain: a randomized clinical trial. JAMA.

[14] Tilbrook HE, Cox H, Hewitt CE, Kang’ombe AR, Chuang L-H, Jayakody S, Aplin JD, Semlyen A, Trewhela A, Watt I (2011). Yoga for chronic low back Pain A randomized trial. Ann Intern Med.

[15] Sherman KJ, Cherkin DC, Wellman RD, Cook AJ, Hawkes RJ, Delaney K, Deyo RA (2011). A randomized trial comparing yoga, stretching, and a self-care book for chronic low back pain. Arch Intern Med.

[16] Wren AA, Wright MA, Carson JW, Keefe FJ (2011). Yoga for persistent pain: new findings and directions for an ancient practice. Pain.

[17] Gatchel RJ, Rollings KH (2008). Evidence-informed management of chronic low back pain with cognitive behavioral therapy. Spine J.

[18] Thorsell J, Finnes A, Dahl J, Lundgren T, Gybrant M, Gordh T, Buhrman M (2011). A comparative study of 2 manual-based self-help interventions, acceptance and commitment therapy and applied relaxation, for persons with chronic pain. Clin J Pain.

[19] DeBar LL, Elder C, Ritenbaugh C, Aickin M, Deyo R, Meenan R, Dickerson J, Webster JA, Yarborough BJ (2011). Acupuncture and chiropractic care for chronic pain in an integrated health plan: a mixed methods study. BMC Complement Altern Med.

[20] Lee AW (2011). Implementation of the veterans health administration national pain management strategy. Transl Behav Medi.

[21] Krebs EE, Becker WC, Zerzan J, Bair MJ, McCoy K, Hui S (2011). Comparative mortality among Department of Veterans Affairs patients prescribed methadone or long-acting morphine for chronic pain. Pain.

[22] Simmonds MJ, Finley EP, Vale S, Pugh MJ, Turner BJ (2015). A qualitative study of veterans on long-term opioid analgesics: barriers and facilitators to multimodality pain management. Pain Med.

[23] Bair MJ, Matthias MS, Nyland KA, Huffman MA, Stubbs DL, Kroenke K, Damush TM (2009). Barriers and facilitators to chronic pain self-management: a qualitative study of primary care patients with comorbid musculoskeletal pain and depression. Pain Med.

[24] Chen JT, Fagan MJ, Diaz JA, Reinert SE (2007). Is treating chronic pain torture? Internal medicine residents’ experience with patients with chronic nonmalignant pain. Teach Learn Med.

[25] Dobscha SK, Corson K, Flores JA, Tansill EC, Gerrity MS (2008). Veterans affairs primary care Clinicians’ attitudes toward chronic pain and correlates of opioid prescribing rates. Pain Med.

[26] Matthias MS, Parpart AL, Nyland KA, Huffman MA, Stubbs DL, Sargent C, Bair MJ (2010). The patient–provider relationship in chronic pain care: providers’ perspectives. Pain Med.

[27] Castiglioni A, Shewchuk RM, Willett LL, Heudebert GR, Centor RM (2008). A pilot study using nominal group technique to assess residents’ perceptions of successful attending rounds. J Gen Intern Med.

[28] Safford MM, Shewchuk R, Qu H, Williams JH, Estrada CA, Ovalle F, Allison JJ (2007). Reasons for not intensifying medications: differentiating “clinical inertia” from appropriate care. J Gen Intern Med.

[29] Brahm C, Kleiner BH (1996). Advantages and disadvantages of group decision-making approaches. Team Performance Management.

[30] Elliott TR, Shewchuk RM (2002). Using the nominal group technique to identify the problems experienced by persons living with severe physical disabilities. J Clin Psychol Med Settings.

[31] Jones J, Hunter D (1995). Consensus methods for medical and health services research. BMJ.

[32] Kizer KW, Jha AK (2014). Restoring trust in VA health care. N Engl J Med.

[33] Kerns RD (2014). Can we improve cognitive–behavioral therapy for chronic back pain treatment engagement and adherence? A controlled trial of tailored versus standard therapy. Health Psychol.

[34] Mehling WE, Price CJ, Daubenmier J, Mike A, Bartmess E, Stewart A (2014). Body awareness and the practice of yoga or meditation in 435 primary care patients with past or current low back pain. J Altern Complement Med.

[35] Cochella S, Bateman K (2011). Provider detailing: an intervention to decrease prescription opioid deaths in Utah. Pain Med.

[36] Buchbinder R (2008). Self-management education en masse: effectiveness of the back pain: Don’t take It lying down mass media campaign. Med J Aust.

[37] Edmond SN, Keefe FJ (2015). Validating pain communication: current state of the science. Pain.

[38] Kroenke K, Krebs EE, Wu J, Yu Z, Chumbler NR, Bair MJ (2014). Telecare collaborative management of chronic pain in primary care: a randomized clinical trial. JAMA.

[39] Dobscha SK, Corson K, Perrin NA, Hanson GC, Leibowitz RQ, Doak MN, Dickinson KC, Sullivan MD, Gerrity MS (2009). Collaborative care for chronic pain in primary care: a cluster randomized trial. JAMA.

[40] Ramirez AG, Turner BJ (2010). The role of peer patients in chronic disease management. Ann Intern Med.

[41] Rosland A-M, Piette JD (2010). Emerging models for mobilizing family support for chronic disease management: a structured review. Chronic Illn.

